# Supplementary figures and images for: Theoretical efficiency limits and speed-efficiency trade-off in myosin motors
Source: PLoS Comput Biol. 2023 Jul 21;19(7):e1011310. doi: 10.1371/journal.pcbi.1011310 (PMC10395908; doi:10.1371/journal.pcbi.1011310)

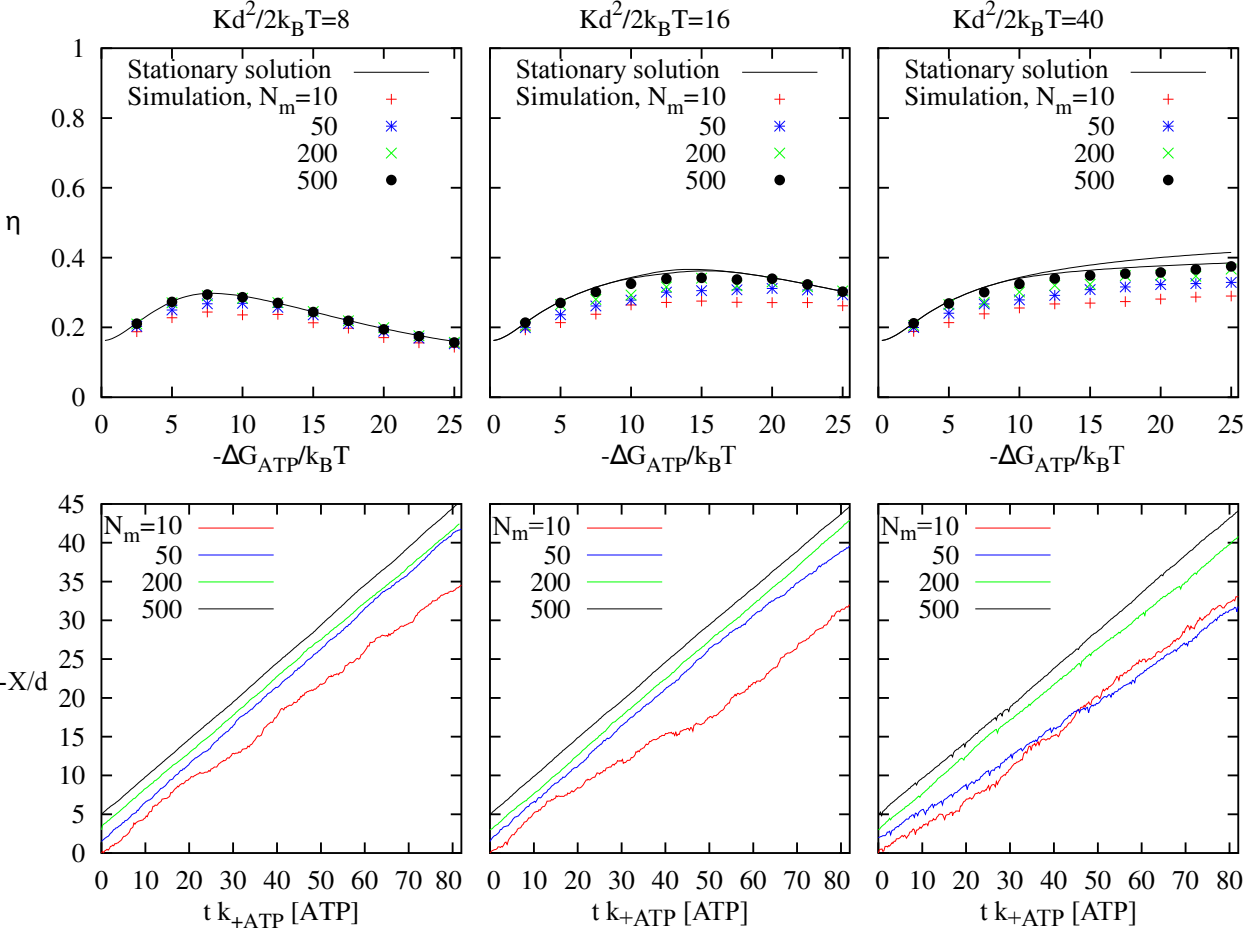

Supplement: S1 Fig — Motors have NB = 2 bound states and parameters obtained from the numerical optimization (Fig 2A in the main text). The upper row shows the simulated efficiency (symbols), compared with the stationary result (main text) for three different values of the dimensionless stiffness 12Kd2/kBT. The bottom row shows example traces (position X vs. time t). (PDF) [file pcbi.1011310.s001.pdf]

$$Kd^2/2k_B T=8$$

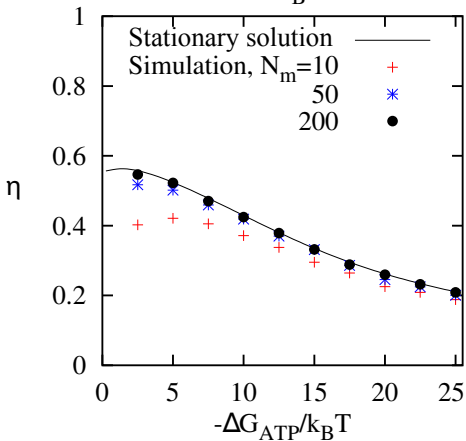

$$Kd^2/2k_B T=16$$

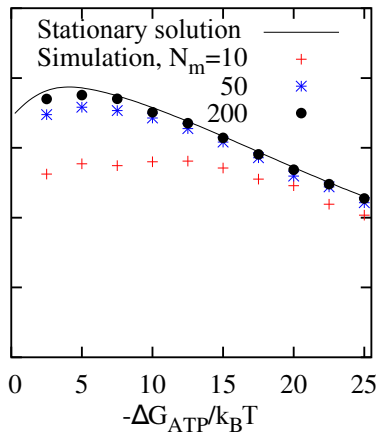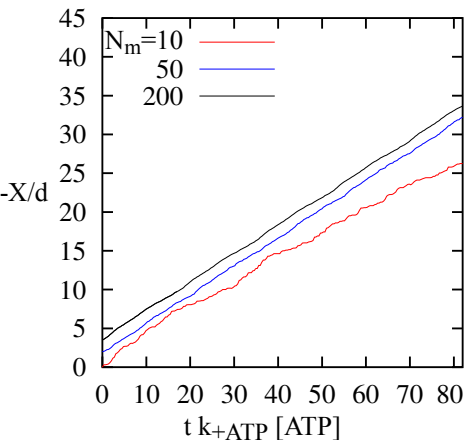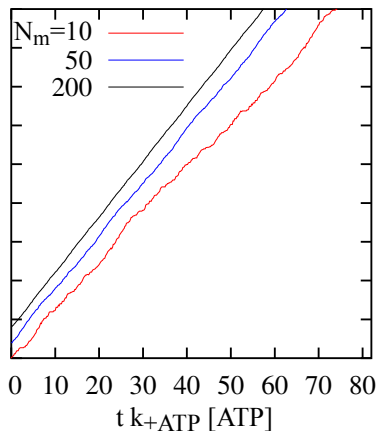

Supplement: S2 Fig — The stationary solutions correspond to dotted lines in Fig 2A in the main text. (PDF) [file pcbi.1011310.s002.pdf]

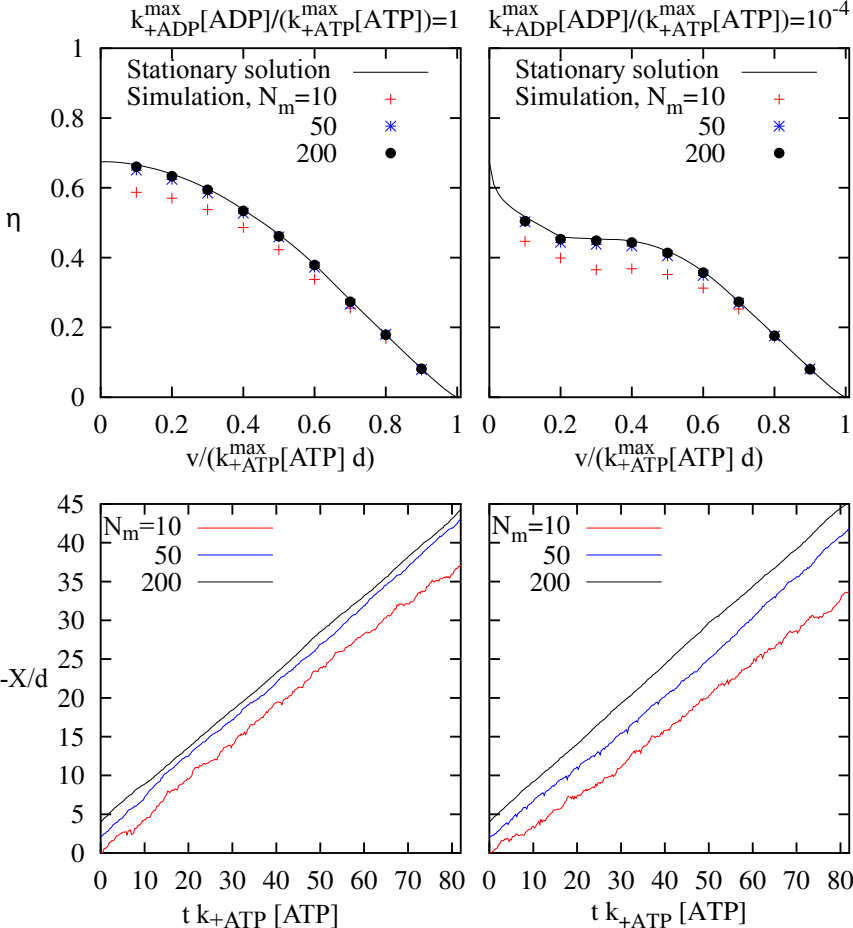

Supplement: S3 Fig — The stationary solution corresponds to solid lines in Fig 3A in the main text. (PDF) [file pcbi.1011310.s003.pdf]

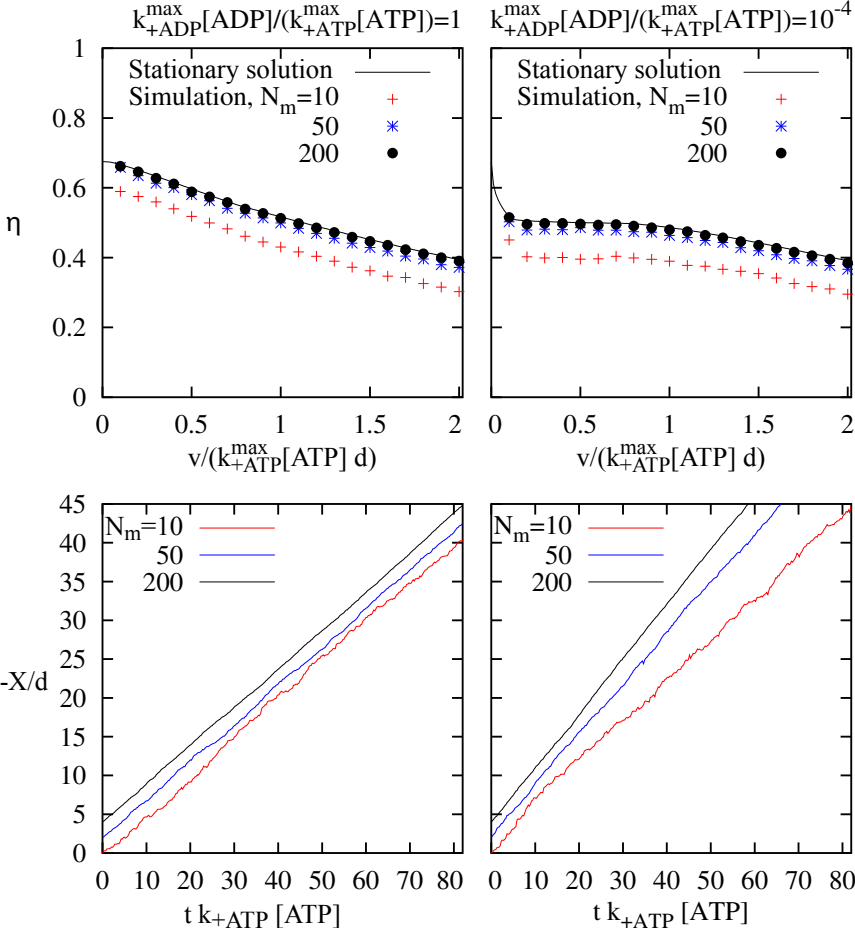

Supplement: S4 Fig — (PDF) [file pcbi.1011310.s004.pdf]

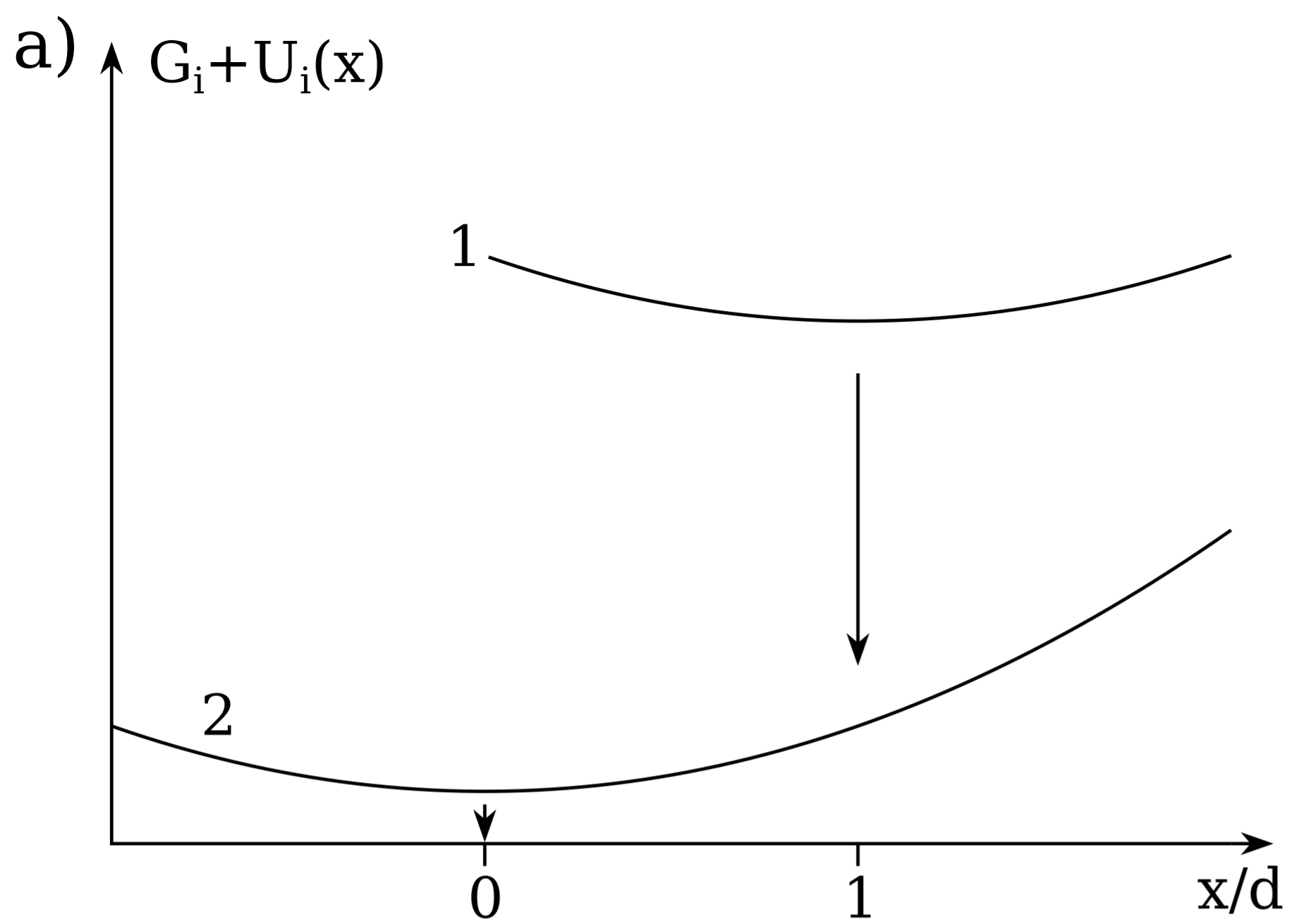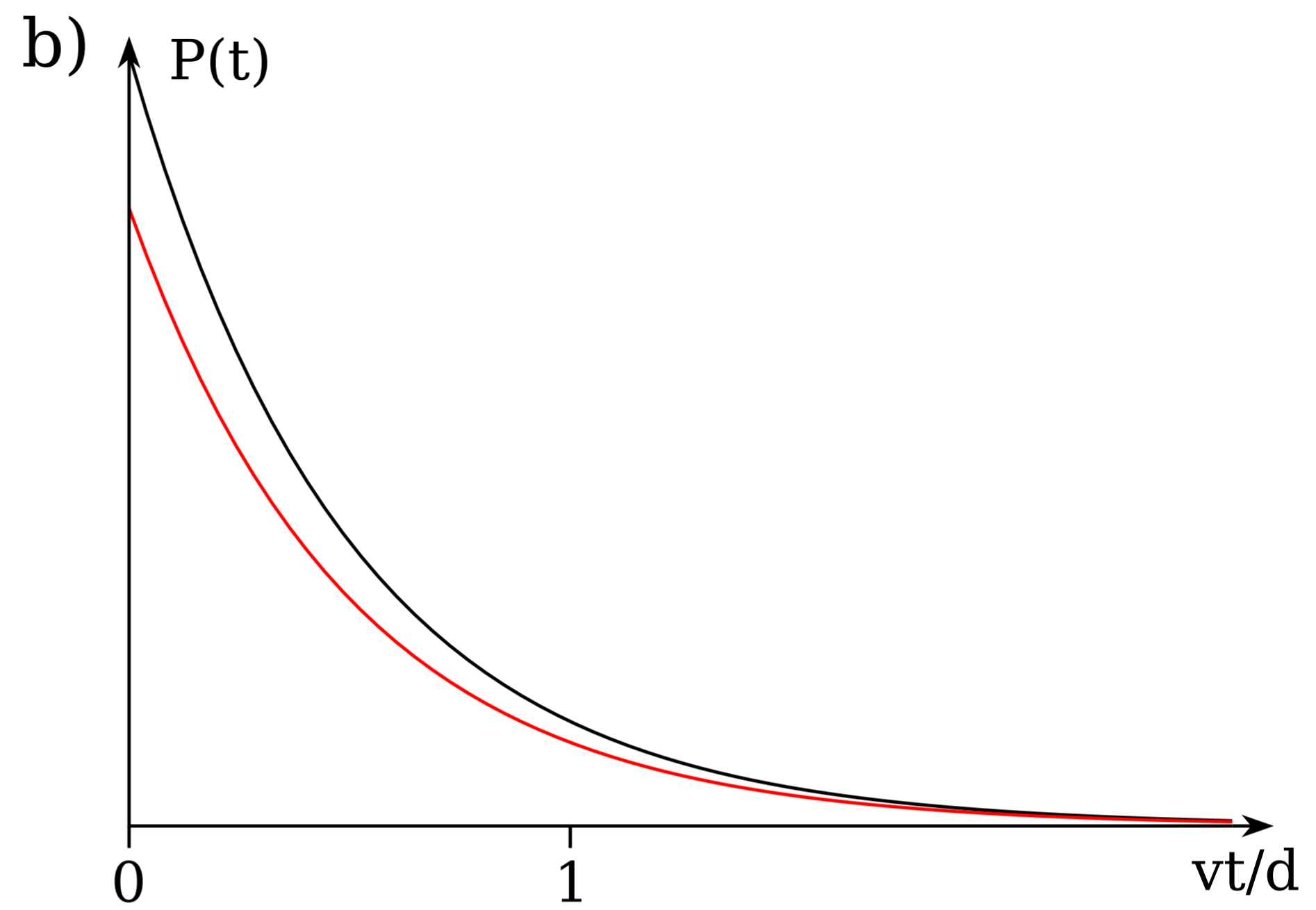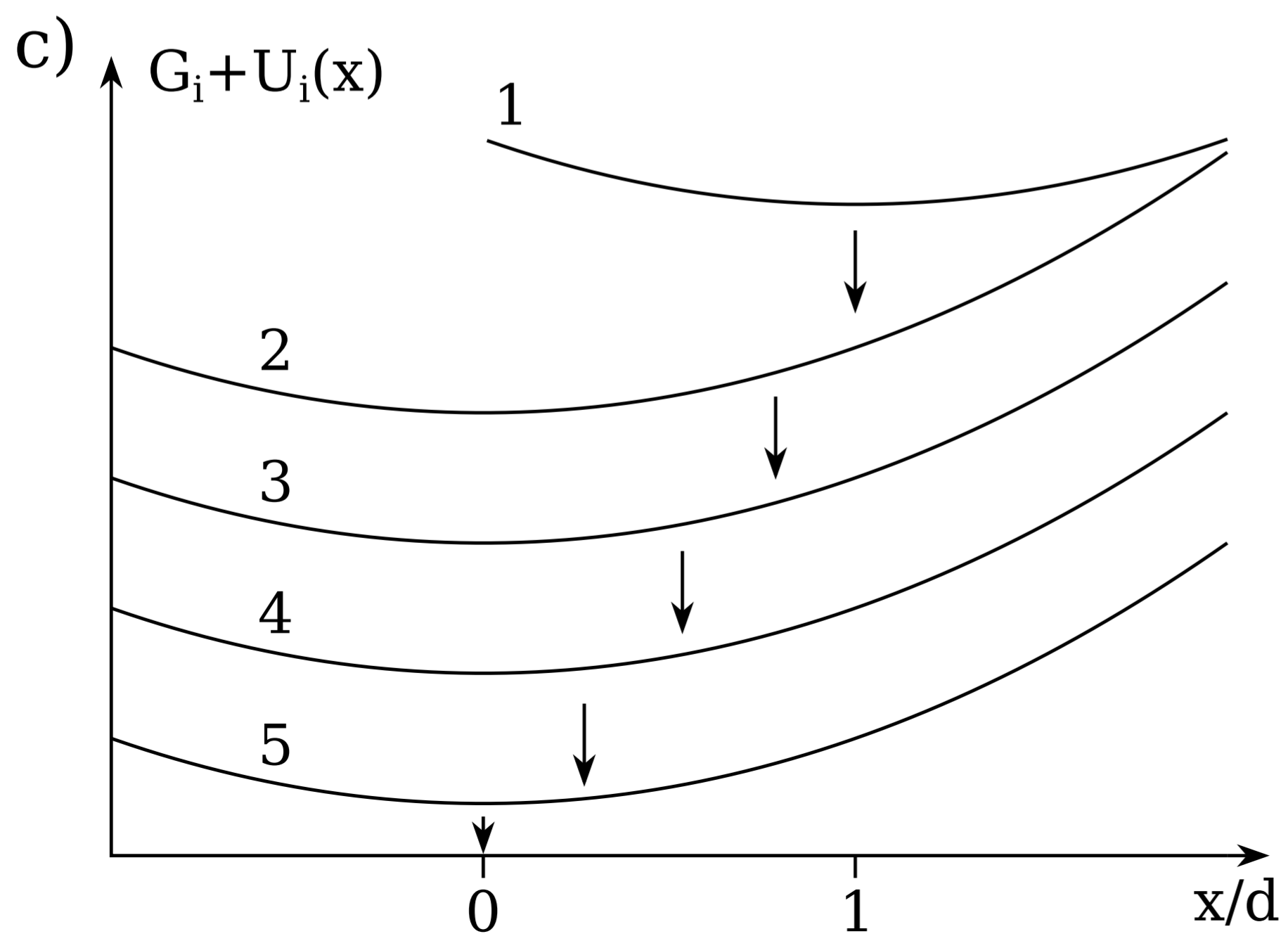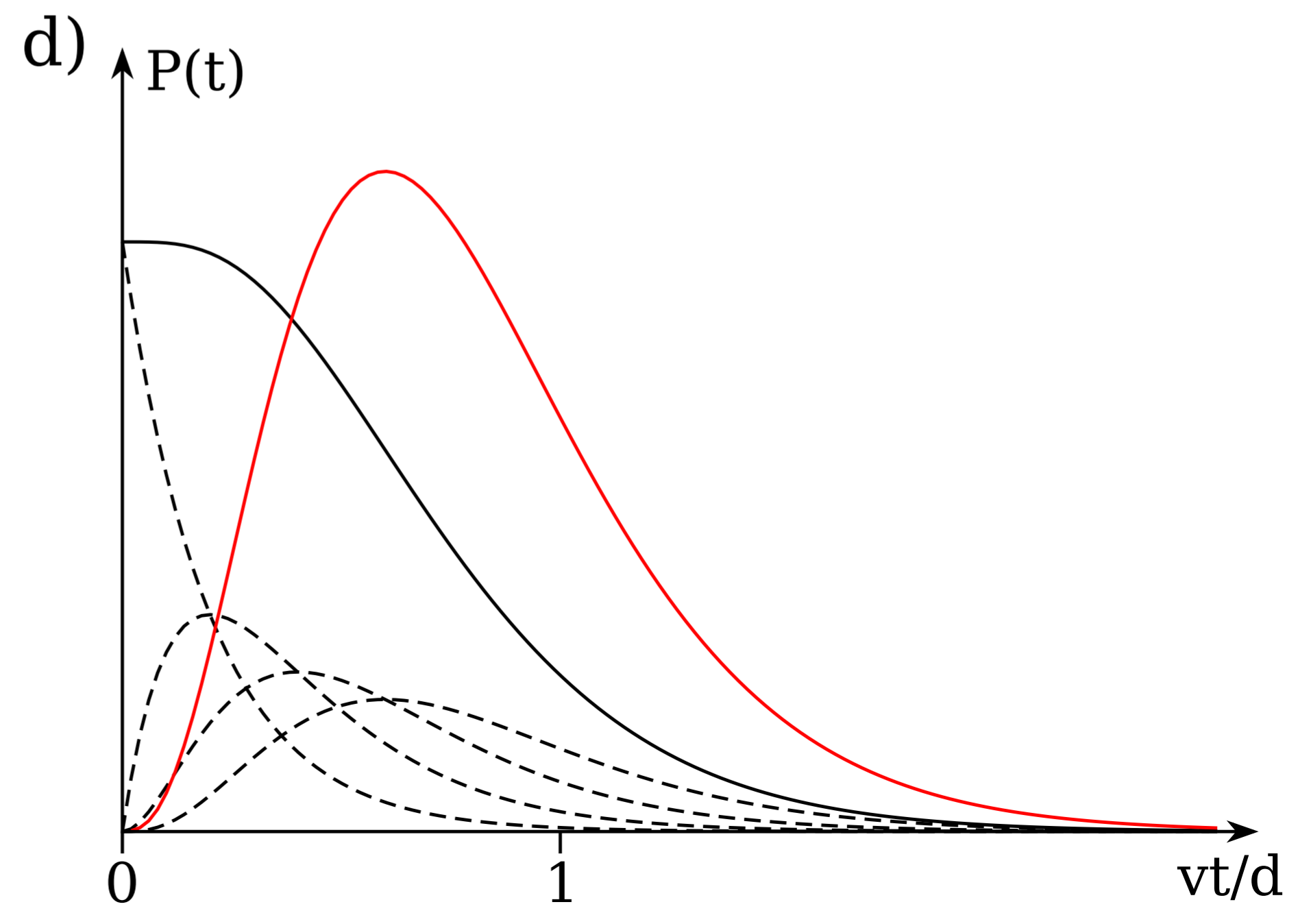

Supplement: S5 Fig — a) Potential landscape with NB = 2 bound states. b) The probability P2(t) to find the motor in state 2 at time t after the attachment (black line). The red line shows the probability density of detachment at time t. c) Potential landscape with NB = 5 bound states. d) The probabilities Pi(t), i = 2, 3, 4, 5 to find the motor in state i at time t after attachment (dashed lines). The solid line shows the total probability P2(t) + P3(t) + P4(t) + P5(t). The red line shows the detachment probability density. The larger number of states narrows the distribution of detaching motors and therefore their elastic energy before detachment. (PDF) [file pcbi.1011310.s005.pdf]
