# Supplementary material for: Theoretical efficiency limits and speed-efficiency trade-off in myosin motors
Source: PLoS Comput Biol. 2023 Jul 21;19(7):e1011310. doi: 10.1371/journal.pcbi.1011310 (PMC10395908; doi:10.1371/journal.pcbi.1011310)
Supplement: Attachment — Submitted filename: ReplyReviewers.pdf [file pcbi.1011310.s008.pdf]

Reviewer #1

We thank the Reviewer for the quick response, for the careful evaluation of our manuscript and for the numerous constructive comments. We have carefully addressed all of them, as seen from the point-by-point response below. Each comment by the Reviewer is shown in blue and our response in black.

1) Page 3, line 66: intuitively, I would have chosen alpha the other way around: detachment rates depend on the strain, but not the attachment rate. Maybe the authors could motivate their choice better.

Detachment rates can clearly be force dependent and there is a large body of literature studying this dependence. The dependence typically has the form exp(|F|d/kT), with a distance parameter d in the range smaller than a 1nm. Note that larger distance parameters, measured for example by Michael Ostap and coworkers, often describe effective detachment rates that include a fast transition before detachment and are therefore not comparable with the elementary detachment rates we use here.

The strain dependence of the attachment rate is an effective one, resulting from the specific geometry at a given time. In order to reach a binding site, the elastic element has to stretch through thermal fluctuations, giving the attachment rate a prefactor of $\exp(-K x^2/2kT)$. At typical distances of a few nm, this term dominates.

We have included a few sentences explaining the choice of alpha=1.

2) Page 4, Fig. 1. caption: I would suggest a different terminology to distinguish between bound states and unbound states. The word “free” state sounds odd to me. “detached state” is also a possibility.

We agree that “unbound” is a more suitable word, so we are now using it consistently.

3) Page 5, Eq. 8, and Eq. 10, the authors should explain that the prime means a spatial derivative and the dot a temporal.

We now explain the meaning of U’ after Eq. 8. We also explicitly state that \dot S is the time derivative of the total entropy.

4) Page 6: Numerical solution: I understand that multi-parametric optimization is computationally difficult, but I would have liked to see a bit more details and discussion about the numerics. How are the initial guesses chosen? How sensitive are the results to the initial guesses? Are there any arguments why the optimized parameter set is not a local extrema, but the global one? Would it be possible to use a different optimization routine to check if the results are consistent?

This information was missing from the original version of the manuscript, but we did in fact use another optimizer, namely an SQP (sequential quadratic programming) routine to determine the optimal solutions outside a hysteresis (thin lines). We now state this in the rewritten methods. In regions of overlap, the two solvers gave the same efficiency. In general, the determination of the optimal efficiency was robust, although there are cases where individual parameters are not uniquely determined (when the Hessian has a zero or close to zero eigenvalue) and therefore show larger variations.

Otherwise, the approach we used was to use a number of different initial parameter values at some points. With a global maximum, we then “tracked” the solutions (i.e., used the optimal parameters from one solution to initialize the next one with the next value of the independent variable) in both directions. Although there were cases where the solver got trapped in a local maximum, these were easy to recognize and replace. We have expanded the description of optimization procedure in Model and Methods.

5) Page 7, Table 1. As a suggestion for the authors, I am wondering if it would be possible to include more information in this table. Maybe by replacing “OPT” with the actual results from one optimization and then printing these numbers either italic or bold and explaining in the caption that these parameters were

optimized. The advantage of such a representation would be to get an idea of the order of magnitude of the values without downloading and opening the zip files.

Making the table more quantitative and informative is definitely a worthy goal. However, the problem we see is that the optimization is always done for a range of values of an independent parameter, like ΔG_{ATP} in Fig. 2a. The majority of parameters that are optimized depend on the independent variable, therefore we think that their presentation (either as tables or as graphs) in the main text is not practical.

In order to give the reader an insight into actual values, as suggested by the Reviewer, we decided to list all parameters used in Figures 2c, 2d, 3c and 3d, which represent a single parameter set each, in the respective captions. We hope that they will serve as a representative sample of parameter values for the reader.

6) Page 8, Fig.2: (a) and (b) label of the axis: eta should be eta^max. Furthermore, the points c and d indicated in figure (a), and (b) could be potentially confusing. Please add a sentence in the caption about those points under (a) and (b), not at the end of the caption.

We agree that η^{max} is a better symbol for maximum efficiency and also that the meaning of the markers should be explained as they appear. We have changed the figures accordingly. We also explain the meaning of dots under panel (a) and do the same for Figure 3 as well.

7) Page 9, line 194: “Although a slow transition leads to entropy production, it can improve the timing of the detachment and thereby reduce larger losses there.” Would it be possible to explain this sentence in more details? It is not clear to me what “improve the timing” means: longer attachment? shorter detached states? improved compared to what? I also don’t understand why an improvement in the timing reduces the losses.

We agree that the statement is too brief and cryptical. We have therefore largely expanded it and it now reads as follows:

“Although a slow transition increases dissipation, a cascade of transitions with similar rate constants narrows the distribution of times between attachment and detachment, i.e., it makes it more deterministic. A narrower distribution means that the elastic energy of strained springs just before detachment, which is another source of dissipation, will be smaller. In the extreme limit of fully deterministic attachment times, it is even possible that all motors detach with exactly zero strain.”

Reviewer #2

We thank the Reviewer for the quick response, for the careful evaluation of our manuscript and for the insightful comments and literature references. We have addressed all of them, as seen from the point-by-point response below. Each comment by the Reviewer is shown in blue and our response in black.

The authors discuss (in the Conclusions) possible limitations e.g. to what extent the results are generally valid, independent of the model used. However, I am not entirely convinced by the arguments. Could you please elaborate?

We have now expanded the section, which reads:

"Besides those constraints, our model makes some further assumptions. For one, we assume a single working cycle without any side branches, for example detachment in the ADP state. Such side branches generally take place between states with a higher free energy difference and therefore increase the dissipation (they can also be seen as leaks in the cycle). On the other hand, we neglect the discrete nature of actin subunits and assume that the myosin heads can bind anywhere on the actin filament. While the effect of discrete binding sites has been estimated as small [], we expect that restricting the sites will somewhat increase the losses of the transition involving initial binding (0->1). The assumptions that the detachment rates are independent of force and that the other transitions depend in the simple way on the free energy difference present a restriction in the space of possible models. Furthermore, like virtually all myosin models, we assume that the stiffness of the elastic element is the same in all states. Also, we do not consider other optimization criteria that may be physiologically relevant, such as the maximum (isometric) force per motor or the ability of motors to work efficiently over a broader range of velocities. Beyond these constraints and assumptions, all parameters are the result of optimization."

The authors also discuss the results in relation to other model studies with general aims, which they claim particularly concern processive motors. It would be great to know which papers the authors refer to in this case. Thus, please cite the relevant papers at this point as a quick look at some of the papers cited in the Introduction does not make it obvious that they do not at all apply to non-processive motors.

We have now included 6 references that study kinesin and F1-F0 from the perspective of stochstic thermodynamics in the conclusions, as suggested. We also rephrased the sentence, to make it clear that the statement that processive motors are better understood refers to theoretical models. Experimentally, simultaneous measurement of ATP hydrolysis and mechanical work at the single molecule level is very challenging, so experimentally the efficiency of non-processive myosins is rather better known.

The lack of reproduction of the experimental efficiency vs velocity plot (Fig. 3; Line 204) seems problematic (if it is really the case?). For experiments see: (e.g. Barclay et al 2010 Prog Biophys Mol Biol). However, I might have misunderstood this; is the velocity here taken as maximum shortening velocity rather than the velocity variation for a given set of parameters vs load (in the force-velocity relationship)? Please clarify!

The Reviewer addresses an important point here which is indeed not well described in the original version of our manuscript. The resolution suggested in the second part of the comment is largely accurate.

The efficiency as a function of the velocity for a fixed set of other parameters has a skewed parabolic dependence with zero in the isometric state, zero at maximum velocity and a maximum inbetween, skewed towards lower velocities. This is the dependence that is measured, for example, in the paper cited by the Reviewer.

What Figure 3 shows is the maximum efficiency that is possible at a given velocity, provided that all parameters (except for the restricted ones, like the second order constant for ATP binding) are optimized for that velocity. These results can only be compared against a variety of isoforms, not the same isoform over a range of contraction speeds. This is also the suggestion proposed by the Reviewer, with the fine difference that the abscissa shows the actual, not the maximum speed.

To address this comment, we expanded Figure 1, panel (d) to show the efficiency as a function of the velocity, calculated for the same example as the force-velocity relationship. Note that in the model with 2 states it is rather symmetric (an inverted parabola), whereas with 3 states it gets a skewed shape, similar to those measured experimentally. We then refer to this figure to stress that the efficiency is always calculated at the peak of this curve, or at the highest point outside a hysteresis if applicable.

The results seem to suggest that slow motors work best if their stiffness is high whereas the opposite applies to fast motors (in addition to non-linearity of the stiffness). The predictions for the magnitude of stiffness seem to be in contrast with the real world data where optical tweezers based experiments (Capitanio et al., PNAS, 2006) have reported lower stiffness for slow than for fast myosin II motors of skeletal muscle.

Our calculation indeed shows that softer motors can be more efficient at high velocities (for harmonic springs) and that the nonlinearity shifts closer to the center for anharmonic springs. We think that stiffness measured 3-bead assays is difficult to compare to myosin in thick filaments. Nevertheless, there are also other papers showing lower stiffness in slow isoforms. To our knowledge, the stiffness measured in those isometric experiments is determined by the pulling part of the elastic element (positive x), therefore not indicative of the possibly softer elasticity in the pulling part. We do not otherwise have an explanation for softer slow isoforms for that from the efficiency perspective. This is now briefly discussed in the revised manuscript.

I wonder if the authors could also extend their discussion to findings in a paper by Barclay (Clin Exp Pharmacol Physiol. 2017) reporting an inverse, linear relationship between maximum normalized power output and efficiency?

We thank the Reviewer for drawing our attention to this interesting result. The complication with this comparison is that the power output is proportional the product of contraction speed and average force per motor (while the volume density of myosin motors is roughly conserved between muscle types). The force per motor again depends on the duty ratio, besides other parameters. We included this result in the discussion, but a quantitative comparison will need a different optimization criterion, which would merit a standalone study.

Minor

Lines 36-37. The definition of power density and force density may not be entirely clear to readers with a background in biology who might find interest in this paper. Please clarify.

Force density is defined as muscle force per cross-section area. For a fixed geometry, it is proportional to the mean force per motor.

The power density is the rate of work per unit volume of muscle. It is proportional to the power per motor, because the volume density of motors is largely conserved.

We have included those explanations as suggested.

Line 131. AFP->AFM. Please spell out “atomic force microscopy” upon first use.

We corrected the typo and also spelled it out.

Legend, Fig. 2. Which are the “thin lines” in panel b. I only find one thin line.

We used the plural, because the blue line also has small region where the two solutions differ by a small amount. It does require a very careful look, though.

Line 178. Could be of value to exemplify “very soft springs” with illustration of the free energy diagrams for such a condition, e.g. in SI.

We have followed the suggestion and included a diagram in the soft-spring regime as supplementary figure S5. In the same figure we also show the distribution of dwell times since initial attachment and show that it becomes narrower in models with more bound states.

Lines 188-190. Based on the linearity in Fig. 2b, the limit of soft springs seems to apply for Kd2/2 up to almost 20 kBT. That does not seem to be consistent with Kd2<...

The analytical arguments in the limit of soft springs provide a simple calculation that gives the exact value of efficiency in the limit of small K and predicts a linear dependence. This argument cannot be reversed, i.e., a linear dependence in itself does not prove that the assumptions of the soft limit are fulfilled. Indeed the numerical optimization shows that the nearly linear dependence extends much further, but an analytical theory in that regime would far exceed the scope of this paper.
